# *Streptococcus mitis/oralis* endophthalmitis management without phakic intraocular lens removal in patient with iris-fixated phakic intraocular lens implantation

**DOI:** 10.1186/1471-2415-14-92

**Published:** 2014-07-15

**Authors:** Jin Kwon Chung, Sung Jin Lee

**Affiliations:** 1 Department of Ophthalmology, Soonchunhyang University Hospital, 59, Daesagwan-gil, Seoul 140-743, Yongsan-gu, Republic of Korea

**Keywords:** Streptococcus mitis/oralis, Endophthalmitis, Iris-fixated phakic intraocular lens, Antibiotic injection

## Abstract

**Background:**

To report a case of *Streptococcus mitis/oralis* endophthalmitis management which had developed after complicated iris-fixated phakic intraocular (pIOL) lens implantation.

**Case presentation:**

A 23-year-old-woman received pIOL implantation followed secondary intraocular intervention to lower intraocular pressure. The patient presented with severe pain and decreased visual acuity and was managed with intravitreal and intracameral antibiotic injection with topical applications of fortified antibiotics. Culture of aqueous humor was positive for *S. mitis/oralis*, which was sensitive to the empiric antibiotic regimen. Clinical features started to improve 5 days after treatment and the pIOL was left in place. The uncorrected distant visual acuity and endothelial cell count were 20/32 and 3143cells/mm^2^ four weeks after treatment, respectively.

**Conclusion:**

*S. mitis/oralis* endophthalmitis after pIOL implantation could be managed with appropriate antibiotic administration without pIOL removal if accompanied by rapid clinical improvement after the initial intensive management in the absence of vitreous involvement.

## Background

Phakic intraocular lenses (pIOLs) are generally accepted as effective and safe treatment options in the correction of moderate to high myopia [1]. Different from LASER assisted vision correction, a pIOL implantation into the anterior or posterior chamber is a reversible operation. This is a strong advantage of pIOL implantation, especially in the context of intra- or postoperative complication. However, intraocular surgery places patients at risk for endophthalmitis, which could lead to permanent visual loss. Although the rate of endophthalmitis is lower in pIOL implantation than in other types of intraocular surgery such as phacoemulsification and posterior chamber (PC) IOL implantation, early diagnosis and proper management is still important in the management of this potentially devastating complication [2,3]. We report a case of infectious endophthalmitis treated successfully without pIOL removal.

## Case presentation

**Figure 1:**
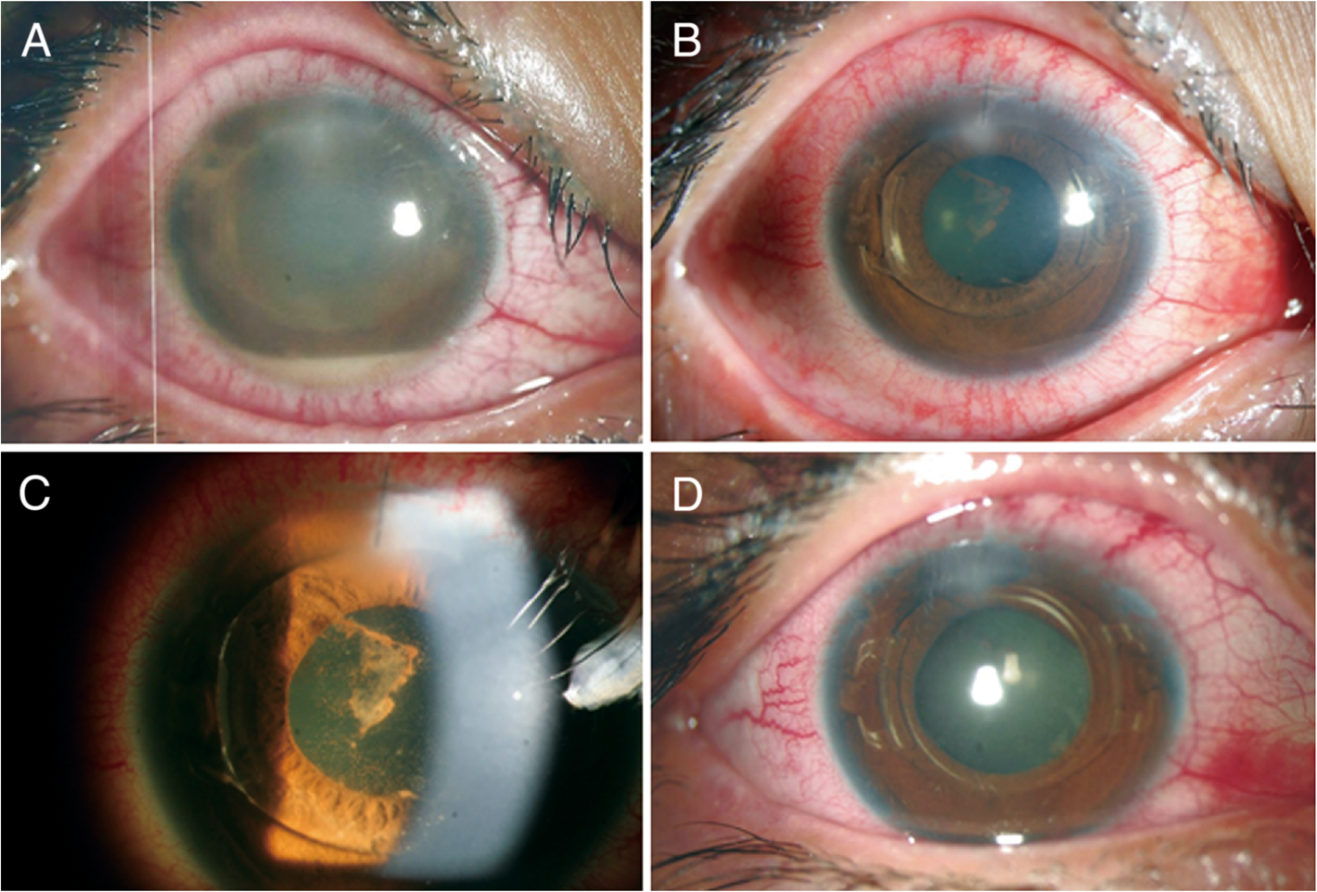
**Anterior segment photographs. A**: Right eye shows conjunctival injection, corneal edema with infiltrates at the corneal incision site, membrane formation around the phakic intraocular lens (pIOL), and a 1.5 mm hypopyon 2 days after implantation. **B**, **C**: The day after anterior chamber irrigation with intracameral antibiotic injection, the eye shows marked decreased inflammation and corneal edema. **D**: At 2 weeks after intravitreal antibiotics injection, uncorrected distant visual acuity improved to 20/40 with a well-centered pIOL.

A 23-year-old-woman was referred to our hospital for severe pain and decreased visual acuity started one day ago in the right eye. Two days prior to this, the patient had foldable iris-fixated pIOL (Artiflex; Ophtec BV, Groningen, the Netherlands) implanted in both eyes at an outside clinic. On postoperative day one, she had undergone anterior chamber (AC) irrigation to remove residual viscoelastics which caused intraocular pressure (IOP) spike in the right eye.On examination, uncorrected distant visual acuity (UDVA) was hand motion with IOP of 21 mmHg for the right eye. Biomicroscopy of the eye revealed severe conjunctival injection, corneal edema, corneal infiltration at superior main incision, membrane formation around the pIOL, and a deep AC with a 1.5 mm hypopyon, which were thought to represent infectious endophthalmitis (Figure 1A). Posterior segment evaluations such as vitreous cell grading and fundus examination were impossible because of severe corneal edema and AC inflammation. B-scan ultrasonography showed no definite vitreous involvement, and the left eye was normal.

Immediate management involved AC irrigation, obtaining aqueous humor for culture and stain, and intravitreal vancomycin (1.0 mg/0.1 cc) and amikacin (0.4 mg/0.1 cc) injection. Gram and KOH stain smear revealed no bacteria or fungus. The patient was also treated with systemic (flomoxef 1.0 g every 12 hours) and topical (fortified vancomycin (50 mg/mL) and amikacin (20 mg/mL) hourly) antibiotics, prednisolone 1.0% four times daily, and homatropine 2% twice daily eye drops for a week, then the frequency was reduced according to the clinical response, culture, and sensitivity results. After 5 days of incubation, cultures became positive for *Streptococcus mitis/oralis*. By day 2 of admission, the patient did not improve so that AC irrigation, intracameral vancomycin (1.0 mg/0.1 cc) and amikacin (0.4 mg/0.1 cc) injection, and subtenon triamcinolone injection (40 mg/1.0 cc) were performed.

After the second round of intervention, the patient began to improve clinically. On day 5, UDVA improved to 20/100, and biomicroscopy revealed moderate AC reaction without hypopyon and decreased inflammatory membrane behind the pIOL (Figures 1B, C). At 2 weeks, UDVA was 20/40 and IOP was 11 mmHg (Figure 1D). Endothelial cell density was measured at 3143cells/mm^2^. At 1 month, UDVA improved to 20/32, and biomicroscopy showed minimal AC reaction and corneal edema.

## Conclusion

Infectious postoperative endophthalmitis is rare but serious complication. Previously, there have been two reported cases of endophthalmitis after iris-fixated pIOL implantation. One case was of an *Aspergillus* endophthalmitis, which was managed with pIOL removal, lensectomty, and anterior vitrectomy with antifungal administration [4]. A second case was caused by *Streptococcus pneumonia* resulting in phthisis bulbi and was managed through therapeutic keratoplasty, removal of the pIOL, lensectomy, and repeated intravitreal injection of antibiotics [5]. For PC pIOL, the rate of endophthalmitis has been reported as approximately 1 case of endophthalmitis per 6000 implantable collamer lens implantation [2]. Oum et al. [6] reported *Pseudomonas* endophthalmitis after PC pIOL implantation. That patient was managed by removal of pIOL, lensectomy, vitrectomy with intravitreal antibiotics injection, and demonstrated CDVA of 20/30 at the end of treatment. To our knowledge, this report represents the first case of infectious endophthalmitis caused by *S.mitis/oralis* after iris-fixated pIOL implantation.

*S. mitis/oralis* is an α-hemolytic gram-positive coccus belonging to the viridians streptococcus group and is commonly found in the oropharynx, gastrointestinal tract, skin, and female genital tract [7]. It is considered to have low virulence and pathogenicity but may cause life-threatening infections, particularly endocarditis and meningitis [7]. Recently, endophthalmitis outbreak of *S. mitis/oralis* after intravitreal injection of bevacizumab has been reported, and contamination during syringe preparation of compounding pharmacy was found as the cause, which implies that microbial contamination could occur at any step of preparation or surgical procedures [8]. The histopathologic finding of patient from this outbreak showed a wide variety of severe pathologic tissue changes and 7 of 12 (58.3%) eyes underwent encucleation or evisceration [9]. Taken together, the virulence and pathogenicity seem to be different on the tissue-by-tissue or organ-by-organ basis.

Our patient had both complicated pIOL implantation and secondary intervention. Complicated, prolonged surgery and repeated intervention are known risk factors for infectious endophthalmitis. The patient in this report achieved relatively favorable UDVA upon treatment, which was different from previously published cases [4]– [6]. *S. mitis/oralis* is less virulent than pathogens previously reported infections endophthalmitis, and the antibiotic sensitivity tests revealed the bacteria to be sensitive to vancomycin and flomoxef used in the treatment. We did not remove the pIOL so that the patient could preserve UDVA. Although there were some differences in surgical procedures, IOL removal is not always recommended for post-cataract surgery endophthalmitis [10]. However, it is usually difficult to identify the source of infection. Moreover, clinical courses vary across individual cases. If the pIOL is suspected to be an infection source or the infection does not appear to clinically improve after intensive initial management, an immediate removal should be considered.

Since AC irrigation, intracameral antibiotics injection and systemic antibiotics administration were not proven to be effective in management of postoperative infectious endophthalmitis in a large prospective studies before, we do not recommend our protocol to all the patients with endophthalmitis after pIOL implantation and further studies with a large sample size should be necessary. Although there was absent of IOP spike in our case, care should be also taken to control IOP after a subtenon injection of a long-acting corticosteroid in a young myopic patient.

Our report illustrates that *S. mitis/oralis* endophthalmitis can develop in patient who underwent complicated iris-fixated pIOL implantation with additional intraocular intervention, and it can be managed with intravitreal and intracameral antibiotics administrations without pIOL removal. The case emphasizes the need for a high level of clinical suspicion during evaluation of patients who have undergone complicated surgery or received additional intraocular intervention.

### Consent statement

Written informed consent was obtained from the patient for publication of this case report and any accompanying images. A copy of the written consent is available for review by the Editor of this journal.

## Competing interests

The authors declare that they have no competing interests.

## Authors’ contributions

JKC: patient interaction, diagnosis, data analysis, and drafting manuscript. SJL: patient interaction, diagnosis, performed surgery, and data analysis. Both authors read and approve the final manuscript.

## Pre-publication history

The pre-publication history for this paper can be accessed here:

http://www.biomedcentral.com/1471-2415/14/92/prepub
